# No significant side‐to‐side differences in retropatellar load distribution more than 7.5 years after isolated MPFL reconstruction: A CT‐osteoabsorptiometry pilot study in nine patients

**DOI:** 10.1002/jeo2.70728

**Published:** 2026-05-04

**Authors:** Lukas Kampik, Friedemann Schneider, Julius Martin Hofner, Rohit Arora, Johannes Dominikus Pallua, Armin Runer

**Affiliations:** ^1^ Department of Orthopaedics and Traumatology Medical University of Innsbruck Innsbruck Austria; ^2^ Department of Sports Orthopaedics, Klinikum Rechts der Isar Technical University of Munich Munich Germany

**Keywords:** CT‐osteoabsorptiometry, MPFL reconstruction, patellofemoral instability, subchondral bone

## Abstract

**Purpose:**

To evaluate long‐term retropatellar load distribution after isolated medial patellofemoral ligament (MPFL) reconstruction using computed tomography‐osteoabsorptiometry (CT‐OAM).

**Methods:**

A retrospective single‐centre pilot study (*n* = 9) was conducted, including patients with isolated MPFL reconstruction with a minimum follow‐up of 7.5 years and without any contralateral knee injury or surgery. Bilateral patellae were analysed using CT‐OAM. Maximum‐intensity projections were subdivided into four patellar regions: medial facet (MF), central ridge (CR), medial portion of the lateral facet (MLF) and lateral portion of the lateral facet (LLF). High‐density area (HDA) fractions were defined as >1000 Hounsfield units (HU). Operated and contralateral patellae were compared using paired Wilcoxon signed‐rank tests with Holm correction and effect sizes were reported as rank‐biserial correlation (*r*). Interregional differences were analysed using Friedman tests followed by pairwise Wilcoxon post hoc comparisons with Bonferroni correction. Sensitivity analyses were repeated using HU thresholds of 900 and 1100.

**Results:**

No significant differences in HDA fractions were detected in any patellar region between the operated and contralateral knee (all adjusted *p* > 0.05; rank‐biserial *r* = 0.02–0.08). Interregional variation was significant (*p* < 0.001), with highest HDA fractions in MLF, followed by LLF, CR and MF. Varying HU thresholds (900 and 1100) yielded similar regional rankings and unchanged inferential outcomes.

**Conclusion:**

At a minimum of 7.5 years after anatomically executed, isolated MPFL reconstruction, CT‐OAM showed no statistically significant side‐to‐side differences in retropatellar subchondral mineralization patterns and the regional distribution remained consistent with physiological loading. While these preliminary findings provide no CT‐OAM‐based evidence of altered long‐term mechanical adaptation or pathological medial overload, the small sample size (*n* = 9) precludes definitive conclusions about equivalence. CT‐OAM demonstrated feasibility of characterizing load‐bearing adaptations after patellofemoral stabilization surgery.

**Level of Evidence:**

Level IV, retrospective case series.

**Abbreviations**
ACL‐RSI: anterior cruciate ligament return‐to‐sport after injury
BMI: body mass index
BPII 2.0: Banff Patellofemoral Instability Instrument 2.0
CDI: Caton–Deschamps index
CR: central ridge
CT: computed tomography
CT‐OAM: computed tomography osteoabsorptiometry
dz: Cohen's dz (paired effect size)
G: gracilis tendon
HDA: high‐density area
HU: Hounsfield units
ICRS: International Cartilage Repair Society
IKDC: International Knee Documentation Committee (Subjective Form)
IQR: interquartile range
LLF: lateral portion of the lateral facet
MF: medial facet
MIP: maximum intensity projection
MLF: medial portion of the lateral facet
MPFL: medial patellofemoral ligament
MPFL‐RSI: medial patellofemoral ligament return‐to‐sport after injury
*n*
: number of subjects
nOP: non‐operated
OP: operated
PROMs: patient‐reported outcome measures
ROM: range of motion
S: semitendinosus tendon
SD: standard deviation
S–G: combined semitendinosus–gracilis tendon
TT–TG: tibial tubercle–trochlear groove distance
VAS: visual analogue scale
*α*
: significance level

## INTRODUCTION

Recurrent patellofemoral instability is a major cause of cartilage damage and a recognized risk factor for patellofemoral osteoarthritis [20, 24, 27]. A systematic review of first‐time traumatic patellar dislocation emphasizes that acute dislocation frequently results in persistent instability, pain, recurrent episodes, reduced sports participation and patellofemoral arthritis [27]. Preventing progressive damage to the patellofemoral joint is therefore a central treatment goal in patients with patellofemoral instability.

The medial patellofemoral ligament (MPFL) is the primary passive restraint against lateral patellar translation in early knee flexion and is disrupted in the majority of acute dislocations [27]. MPFL reconstruction has become the standard surgical treatment when conservative management fails and frequently restores stability [26]. However, biomechanical cadaver and computational studies have demonstrated that small deviations in femoral tunnel position or excessive graft tensioning can lead to overconstraint of the patella [23, 28, 30]. This condition increases medial patellofemoral contact pressures, alters the native kinematic pattern and may create non‐physiological loading conditions despite clinically successful stabilization [18, 21]. These findings raise the question of whether an anatomical MPFL reconstruction truly restores a physiological long‐term loading pattern at the patellofemoral joint.

While acute biomechanical alterations following MPFL reconstruction have been well characterized in cadaveric and computational models, the chronic effects on subchondral bone remodelling, reflecting cumulative in vivo loading over years, remain poorly understood. Computed tomography osteoabsorptiometry (CT‐OAM) allows visualization of subchondral mineralization patterns that reflect the long‐term loading history and mechanical adaptation of individual joints [19]. It serves as an imaging surrogate of chronic subchondral joint loading rather than a diagnostic tool for osteoarthritis.

Therefore, the aim of this study was to compare subchondral mineralization patterns of operated and contralateral patellae at a minimum follow‐up of 7.5 years after isolated MPFL reconstruction using CT‐OAM. The hypothesis was that subchondral mineralization patterns would not differ significantly between operated and contralateral patellae.

## MATERIALS AND METHODS

### Study design

This retrospective, single‐centre study was conducted at the Department of Orthopaedics and Traumatology of the Medical University of Innsbruck. Ethical approval was granted by the institutional review board (EK Nr: 1114/2021). The study complied with the Declaration of Helsinki and investigated long‐term retropatellar loading patterns after MPFL reconstruction using CT‐based osteoabsorptiometry derived from standard clinical bone CT data. Given the limited sample size and exploratory nature of this investigation, the study was designed as a proof‐of‐concept analysis rather than a powered confirmatory trial.

### Patients

Patients who had undergone MPFL reconstruction with autologous tendon between June 2009 and December 2014 were screened. Inclusion criteria were: signed informed consent, isolated MPFL reconstruction after clinically and radiologically confirmed isolated MPFL rupture, 5 years postoperative follow‐up. Exclusion criteria were: patellofemoral chondromalacia ≥ International Cartilage Repair Society (ICRS) [2] grade IIIb prior to surgery, age < 18 years at time of inclusion, ipsilateral knee interventions except the isolated MPFL reconstruction, contralateral knee interventions, metallic implants or pacemakers. Patients with high‐grade patellofemoral risk factors like tibial tuberosity–trochlea groove (TT–TG) distance > 25 mm, patella alta (Caton–Deschamps index [CDI] > 1.3), severe trochlear dysplasia (Dejour C and D) and severe valgus malalignment >5° were also excluded. Nine patients met all criteria, consented to participation and were included in the clinical and radiological follow‐up. All included patients had achieved a minimum follow‐up of 7.5 years at the time of study execution, providing adequate time for chronic subchondral bone remodelling in response to altered patellofemoral loading patterns.

### Surgical techniques

Anatomic MPFL reconstruction was performed using autologous hamstring grafts. After diagnostic arthroscopy to rule out intra‐articular pathologies, the gracilis tendon was harvested in standard fashion. The medial border of the proximal patella was exposed through a small longitudinal incision, and the patellar fixation site was chosen at the junction of the proximal third and the middle third of the medial patellar edge, corresponding to the native MPFL insertion zone. The graft was then secured to the medial patellar border using knotless suture anchors (SwiveLock® or PushLock®, Arthrex), ensuring fixation along the anatomical footprint. The graft was routed between the second and third capsular layers and fixed at the femoral insertion using fluoroscopic identification of the anatomic MPFL insertion [25]. Femoral fixation was performed with an interference screw, with the knee flexed at approximately 20°–30° and the patella slightly lateralized to avoid overtensioning of the graft. All reconstructions were performed using a standardized surgical technique, consistent tensioning methods and a uniform postoperative rehabilitation regimen. The standardized rehabilitation protocol included physical therapy with cryotherapy, manual lymphatic drainage and isotonic strengthening exercises for the quadriceps starting on the second postoperative day. A gradual progression from partial to full weight‐bearing was allowed in the first three postoperative weeks, depending on the subjective pain level. From Week 7 onward, further stabilization and proprioception training were recommended. Full return to physical activity was allowed after 4–6 months.

### Radiological follow‐up and CT imaging

Follow‐up imaging included three‐plane radiographs and bilateral knee CT of the affected knee. Three plane and full‐leg radiographs were used to assess patellar height (CDI), degree of osteoarthritis according to the Iwano classification [12] and mechanical limb alignment. Bilateral CT imaging was performed using a Siemens SOMATOM Drive scanner (Siemens Healthineers, Siemens) to enable intraindividual subchondral bone density distribution using CT‐OAM. Acquisition parameters: 120 kV, low‐dose protocol, 0.7 mm slice thickness, table feed = 10 mm, sharp kernel (Siemens Br62s) reconstruction. Patients were positioned supine with knees extended. Images were exported in DICOM format and processed in Analyze 15.0 (AnalyzeDirect Inc.) for segmentation and image analysis.

On postoperative CT, standardized true lateral reconstructions were generated by superimposing the posterior femoral condyles. The femoral tunnel reference point was defined as the cortical entry point at the medial femoral cortex. A coordinate system consisting of a line tangent to the posterior femoral cortex and a perpendicular line through the posterior origin of the medial femoral condyle was applied [11]. Anterior‐posterior and proximal‐distal coordinates, as well as the Euclidean distance to the predefined anatomical femoral reference location, were measured. Anatomical placement was defined as a deviation of <5 mm [7].

### Patient‐reported outcome measures (PROMs) and clinical examination

Validated knee‐specific PROMs were collected: Lysholm Knee Score [17], the Tegner Activity Scale [29, 31], the Kujala Anterior Knee Pain Scale [14], the International Knee Documentation Committee (IKDC) Subjective Form [10] and the Banff patellofemoral Instability Instrument 2.0 (BPII 2.0) [15]. Pain intensity was rated on a visual analogue scale (VAS). Higher values indicate better function for Lysholm, Kujala, IKDC and BPII 2.0, and higher activity for Tegner, and greater pain for VAS. Psychological readiness to return to sport was assessed using an MPFL‐RSI score adapted from the anterior cruciate ligament return‐to‐sport after injury (ACL‐RSI) [32], acknowledging the absence of a patellofemoral‐specific validation [16]. All patients underwent standardized bilateral knee examination focusing on patellar tracking and instability. Clinical examination included assessment of axis alignment, rotation and patella height, as well as functional tests of the extensor mechanism. Patellar instability‐specific tests included apprehension test, patellar tilt test, Zohlen test and J‐sign assessment.

### CT‐OAM and image analysis

**Figure 1:**
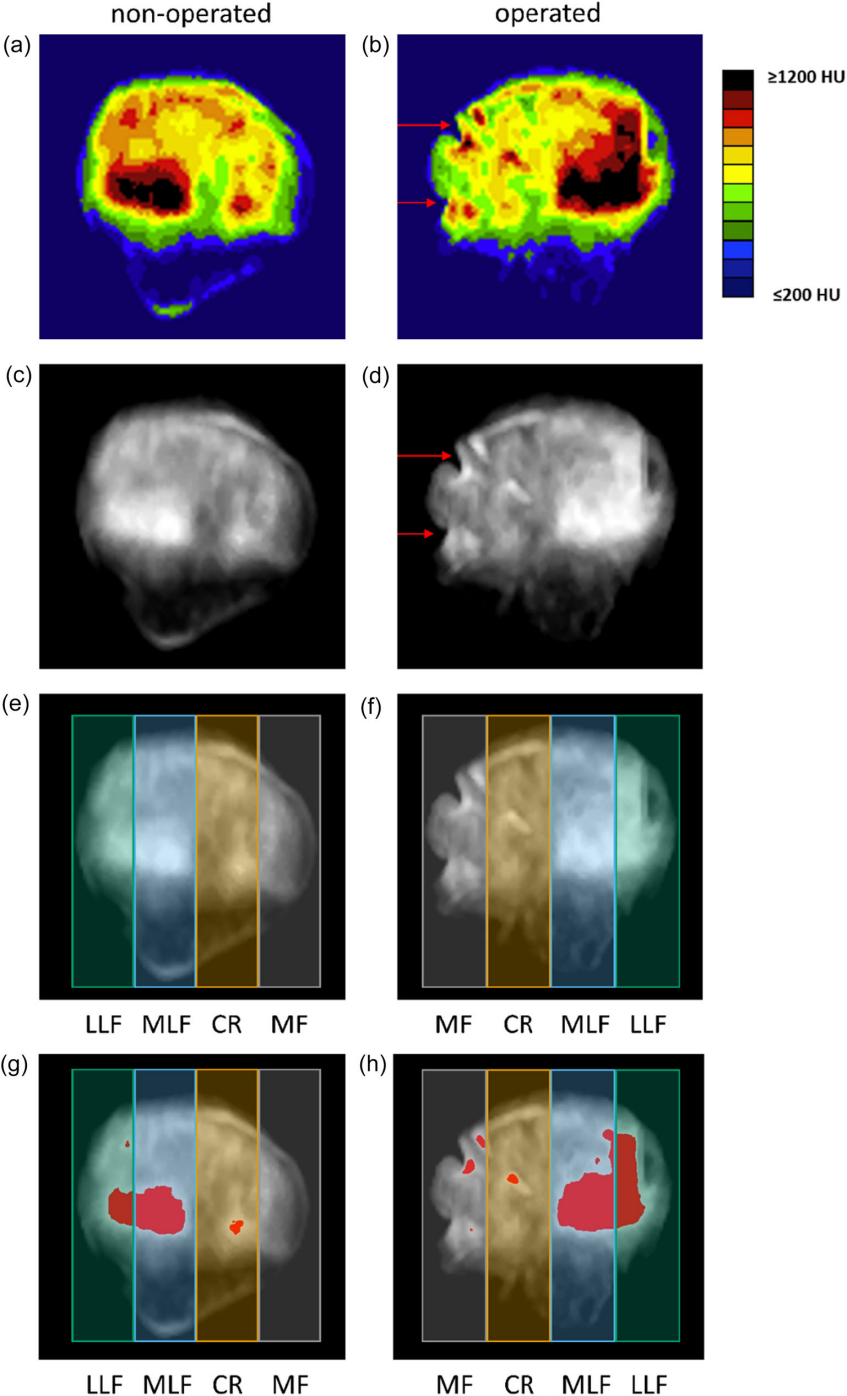
Exemplary visualization of subchondral mineralization and HDA distribution in a contralateral (left column) and operated patella (right column). (a, b) Colour‐coded HU maximum intensity projections (MIPs). Red arrows indicate focal cortical irregularities along the patellar margin. (c, d) Corresponding grayscale MIPs, highlighting cortical irregularities (red arrows). (e, f) Stripe subdivision: MF, CR, MLF, LLF. (g, h) HDA overlay (HU > 1000). CR, central ridge; HDA, high‐density area; HU, Hounsfield units; LLF, lateral portion of the lateral facet; MF, medial facet; MLF, medial portion of the lateral facet.

CT‐OAM is based on voxelwise CT attenuation values that reflect long‐term mineralization. After segmentation, maximum intensity projections (MIPs) were generated using a semi‐automated workflow: The projection axis was manually defined for one reference patient (right patella of P01) to approximate an orthogonal view to the articular surface. All other patellae were then registered to this reference orientation using ANALYZE 15.0 (AnalyseDirect, Inc.). Left‐sided patellae were temporarily flipped on the *x*‐axis to match right‐sided orientation, ensuring consistent anatomical correspondence across all images. Left‐sided patellae were flipped back to their original orientation, ensuring that all subsequent regional labelling corresponded to anatomical positions. A density window of 200–1200 Hounsfield units (HU) was applied to exclude soft‐tissue noise and to include the full mineralization range of subchondral bone. The resulting MIPs were first visualized using a standardized colour map to illustrate the spatial distribution of subchondral density. Each MIP was additionally exported as an 8‐bit grayscale image (0–255), in which higher grayscale values correspond to higher HU, representing denser subchondral bone according to Wolff's law [22, 23, 24]. The density window (200–1200 HU) was linearly mapped to the 8‐bit grayscale range (0–255). All subsequent image analysis and statistics were performed in R (Version 4.4.2; R Core Team) using RStudio (2024.12.1 Build 563; © 2009–2025 Posit Software, PBC). A customized method was developed to standardize regional subdivision. For each patella, vertical boundary lines were positioned at the medial and lateral edges of the patella to define a rectangular bounding box spanning from edge to edge. Within these bounding boxes, four equally wide vertical stripes were automatically cut. The stripes were labelled by approximate anatomical location, in line with prior CT‐OAM reports [13], as medial facet (MF), central ridge (CR), medial portion of the lateral facet (MLF) and lateral portion of the lateral facet (LLF) from medial to lateral, with reversed image‐space labelling for left patellae to ensure anatomical correspondence (see Figure 1). This proportional subdivision ensured equal‐width regions relative to each individual patella and enabled direct anatomical comparison between the operated and contralateral sides. For each strip region, the fraction of high density area (HDA) was computed as

$$\mathrm{HDA}\;\mathrm{fraction}=\;\frac{\mathrm{Pixels}>\mathrm{Threshold}\;\mathrm{HU}\;\mathrm{in}\;\mathrm{region}}{\mathrm{Pixels}\;>\;\mathrm{Threshold}\;\mathrm{HU}\;\mathrm{in}\;\mathrm{entire}\;\mathrm{patella}},$$

where the denominator represents the total number of pixels exceeding the threshold across all four regions combined. A threshold of 1000 HU was used to define HDA, representing regions of increased subchondral mineralization associated with chronic mechanical loading [4, 13]. All HDA thresholds (900, 1000 and 1100 HU) were applied to grayscale values (198, 204 and 208, respectively) after linear mapping of the 200–1200 HU window to the eight‐bit range (0–255). To evaluate robustness, additional thresholds of 900 and 1100 HU were analysed in a sensitivity analysis.

### Statistical analysis

Statistical analyses were performed in R (Version 4.4.2, R Core Team) using RStudio (2024.12.1 Build 563, © 2009‐2025 Posit Software, PBC). Data distribution was assessed using the Shapiro–Wilk test. Because the data were non‐normally distributed and the design was paired (operated vs. contralateral within the same subject), Wilcoxon signed‐rank tests were applied for each region. *p* Values were adjusted for multiple comparisons using the Holm correction. Effect sizes were reported as rank‐biserial correlation (*r*) for paired comparisons. Because HDA in CT‐OAM depends on the selected HU threshold and no universally accepted cut‐off exists, a sensitivity analysis was performed. All statistical comparisons were therefore repeated using HU thresholds of 900, 1000 and 1100 to confirm that side‐to‐side relationships and regional patterns were stable and not driven by threshold choice. Interregional comparisons, independent of surgical status, were performed using Friedman tests (non‐parametric repeated‐measures analysis of variance) to account for the within‐subject clustering of paired knees. For each patient, HDA fractions were averaged across both operated and contralateral knees per region, and the four patellar regions (MF, CR, MLF, LLF) were compared as repeated measures. Post‐hoc pairwise comparisons were performed using Wilcoxon signed‐rank tests with Bonferroni correction. Statistical significance was set at *α* = 0.05 (two‐sided).

## RESULTS

### Patient's assessment and follow‐up

Nine patients (*n* = 7 females, 2 males) with unilateral, isolated MPFL reconstruction were included (Table 1). Mean age at surgery was 20.7 ± 4.5 years (range: 14–27), with mean postoperative follow‐up at imaging of 9.7 ± 1.6 years (range: 7.7–12.3). Retropatellar cartilage lesions (ICRS classification [10, 24]) were present in *n* = 3 cases. No patient underwent revision surgery or showed relevant patellofemoral osteoarthritis (Iwano et al. [12]). All femoral tunnels were classified as anatomically positioned.

**Table 1:** Patient demographics and surgical details, including sex, age at time of surgery, operated side, type of graft used for MPFL reconstruction and fixation techniques at the patella, femoral insertion sites, retropatellar cartilage lesion grade (ICRS classification) and anatomical femoral tunnel position [7, 11].

Patient ID	Sex	Age at surgery	BMI (kg/m^2^)	Operated side	Graft type	Patellar fixation	Follow‐up (years)	Trochlea (Dejour grade)	TT–TG	Tilt	CDI	Retropatellar cartilage lesion (ICRS grade)	Postoperative subluxation	Anatomical femoral tunnel position
P1	Female	17	24.2	Right	G	SwiveLock®	12.3	None	18.5	29.2	1.2	0	No	Yes
P2	Female	27	20.8	Right	G	SwiveLock®	10.4	A	5.9	14.4	1.2	0	Yes	Yes
P3	Female	20	20.4	Left	G	SwiveLock®	8.3	None	7.6	16.8	1.3	0	No	Yes
P4	Female	26	24.0	Right	G	Not specified	8.9	None	9.6	1.9	1.0	0	No	Yes
P5	Female	14	29.0	Right	G	SwiveLock®	8.3	A	17.6	17	1.2	0	No	Yes
P6	Male	25	23.8	Left	S–G	Not specified	11.3	B	6.5	15.5	1.1	0	No	Yes
P7	Female	20	23.5	Left	G	SwiveLock®	9.0	None	7.7	15.5	1.2	I	No	Yes
P8	Female	17	32.1	Left	S	SwiveLock®	11.0	None	9.0	13.1	1.0	II	Yes	Yes
P9	Male	20	26.6	Right	G	SwiveLock®	7.7	A	9.7	18.9	1.2	II	No	Yes

Abbreviations: BMI, body mass index; CDI, Caton–Deschamps Index; G, gracilis tendon; ICRS, International Cartilage Repair Society; MPFL, medial patellofemoral ligament; none, no trochlear dysplasia; S, semitendinosus tendon; S–G, semitendinosus–gracilis tendon; TT–TG, tibial tubercle–trochlear groove distance.

### Clinical and functional outcomes

PROMs were available for seven patients. Median scores were high across all instruments (Table 2). No recurrent patellar dislocations were reported by the patients. Two patients reported lateral subluxation. Apprehension and Zohlen tests were negative in all cases. A positive J‐sign occurred in *n* = 2, and medial tenderness in one patient. Anterior knee pain occurred *n* = 2. Mild crepitations were observed in *n* = 4 patients (median 0, interquartile range [IQR] 0–1; 0 = none, 1 = mild crepitation, 2 = crepitation with mild pain, 3 = crepitation with pain > mild). Full extension was achieved in all cases, median flexion was 140° (IQR 140–150). Overall, joints were clinically stable with preserved range of motion.

**Table 2:** PROMs at postoperative follow‐up after isolated MPFL reconstruction.

Variable	*n*	Median [IQR]
VAS 0–10	7	4.0 [2.5–4.0]
Lysholm Score	7	85.0 [85.0–95.0]
Tegner Activity Scale	7	4.0 [4.0–5.0]
Kujala Score	7	92.0 [83.5–96.5]
IKDC 2000 Score (%)	7	82.0 [79.5–87.0]
BPII 2.0 Score (%)	7	92.0 [71.0–93.0]
MPFL RSI Score	7	90.0 [58.5–95.0]

*Note*: Values are presented as median [IQR]. Higher scores indicate better function for Lysholm, Kujala, IKDC, BPII 2.0 and MPFL‐RSI; higher activity for Tegner; and greater pain intensity for VAS.

Abbreviations: BPII 2.0, Banff Patellofemoral Instability Instrument 2.0; IKDC, International Knee Documentation Committee Subjective Form; IQR, interquartile range; MPFL, medial patellofemoral ligament; MPFL‐RSI, medial patellofemoral ligament return‐to‐sport after injury; *n*, number of patients with available data; PROMs, patient‐reported outcome measures; VAS, visual analogue scale.

### CT‐OAM

On visual inspection, mineralization maxima were predominantly located in the MLF region. In operated patellae, maxima were localized in the MLF region in *n* = 6, in the LLF region in *n* = 2 and were indeterminate in *n* = 1. In contralateral patellae, maxima were located in the MLF region in *n* = 7, in the LLF region in *n* = 1 and were indeterminate in *n* = 1. Quadrant analysis showed inferolateral localization of the HDA in *n* = 7 operated and *n* = 7 contralateral patellae, superolateral localization in *n* = 1 on each side and indeterminate findings in *n* = 1 per side. Contour irregularities along the patellar margin were visible in *n* = 7 operated patellae, consistent with prior surgical intervention. Figure 1 provides a representative example illustrating inferolateral density maxima and postoperative contour alterations. All CT‐OAM images used for visual inspection are provided in Supporting Information S1: Figure 1.

### Comparison of operated and contralateral patellae

**Figure 2:**
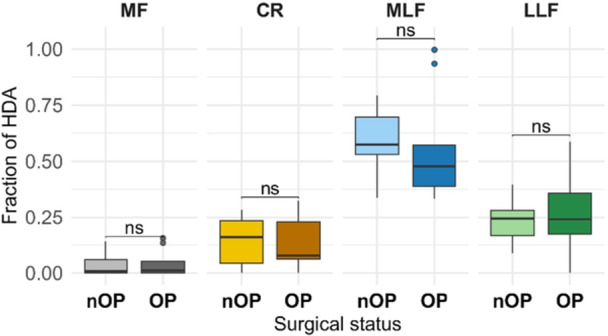
Comparison of HDA fractions (>1000 HU) between operated and contralateral nOP patellae across different patellar regions (MF, CR, MLF, LLF). Boxplots show the distribution of HDA fractions per region. No statistically significant differences were observed between operated and nOP sides (Wilcoxon signed‐rank, all adjusted *p* > 0.05). CR, central ridge; HDA, high‐density area; HU, Hounsfield units; LLF, lateral portion of the lateral facet; MF, medial facet; MLF, medial portion of the lateral facet; nOP, non‐operated; ns, not significant; OP, operated.

No statistically significant side‐to‐side differences were detected between operated and contralateral non‐operated patellae (all Holm‐adjusted *p* = 1; rank‐biserial *r*: 0.02–0.08) (Table 3, Figure 2). Individual patient‐level data demonstrating inter‐individual variability are provided in Supporting Information S1: Table 1 and Figure 2.

**Table 3:** Median [IQR] of HDA fractions for each patellar region (MF, CR, MLF, LLF), shown separately for operated and contralateral nOP sides, as well as combined across both knees.

Region	Surgical status	Median (%)	IQR (%)	*p*‐adjusted	Rank‐biserial *r*
MF	nOP	0.8	0–6.0	1	0.08
	OP	1.1	0–5.2		
	Total	1.0	0–5.8		
CR	nOP	16.0	4.3–23.4	1	0.04
	OP	7.8	6.2–22.8		
	Total	11.9	4.8–23.2		
MLF	nOP	57.5	53.0–69.7	1	0.02
	OP	47.9	38.9–57.2		
	Total	53.5	40.5–67.1		
LLF	nOP	24.5	16.7–28.2	1	0.06
	OP	24.1	17.3–35.9		
	Total	24.3	16.8–34.3		

*Note*: Holm‐adjusted paired *p* values (Wilcoxon signed‐rank) reported once per region. Effect size (rank‐biserial correlation, *r*) refers to these side‐to‐side comparisons. ‘Total’ values (combined operated and nOP) were used for interregional analysis (Friedman test with Bonferroni‐adjusted Wilcoxon signed‐rank post hoc tests).

Abbreviations: CR, central ridge; HDA, high‐density area; IQR, interquartile range; LLF, lateral portion of the lateral facet; MF, medial facet; MLF, medial portion of the lateral facet; nOP, non‐operated; OP, operated; *p*‐adjusted, Holm‐adjusted *p* value.

### Sensitivity analysis (HU thresholds)

Varying HU thresholds (900, 1100) yielded similar regional patterns and statistical outcomes (Supporting Information S1: Table 2).

### Regional variation in HDA fractions

**Figure 3:**
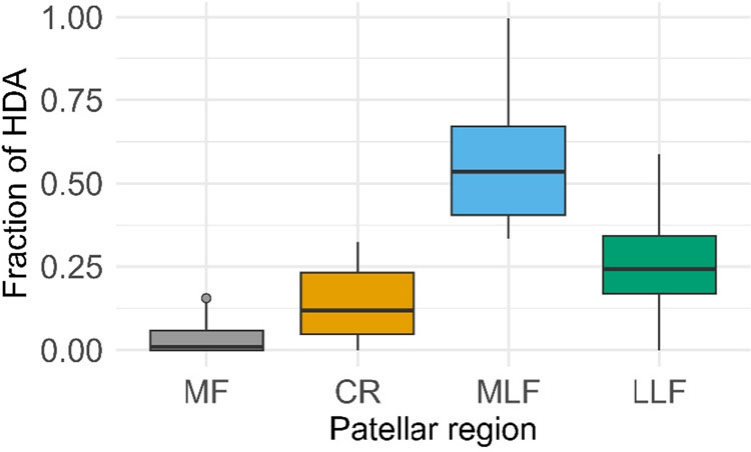
Distribution of HDA fractions across patellar stripe regions. Boxplots illustrate region‐specific differences in subchondral mineralization. CR, central ridge; HDA, high‐density area; LLF, lateral portion of the lateral facet; MF, medial facet; MLF, medial portion of the lateral facet.

Friedman tests revealed significant inter‐regional differences (*χ* ^2^(3) = 21.13, *p* < 0.001), with MLF showing the highest HDA fractions, followed by LLF and CR, and MF (Tables 3 and 4, Figure 3).

**Table 4:** Pairwise comparisons of patellar strip regions based on post hoc comparison (Wilcoxon signed‐rank) following a significant Friedman result.

Compared strip region	*p*‐adjusted
CR–LLF	1
CR–MF	0.07
LLF–MF	0.023
CR–MLF	0.023
LLF–MLF	0.047
MF–MLF	0.023

*Note*: Bonferroni‐adjusted p‐values are reported for all pairwise comparisons. Significant differences (*p* < 0.05) are observed between most strip combinations.

Abbreviations: CR, central ridge; HDA, high‐density area; LLF, lateral portion of the lateral facet; MF, medial facet; MLF, medial portion of the lateral facet; *p*‐adjusted, bonferroni‐adjusted *p*‐value.

## DISCUSSION

The most important finding of this study is that subchondral mineralization patterns did not differ between operated and contralateral patellae >7.5 years after isolated MPFL reconstruction. No statistically significant side‐to‐side differences were detected in any patellar region (all adjusted *p* > 0.05; rank‐biserial *r*: 0.02–0.08), and both sides maintained an inferolateral‐dominant distribution pattern. This physiological loading pattern, characterized by higher mineralization in the lateral facets compared with the CR and MF, corresponds to the expected retropatellar loading pattern and indicates no CT‐OAM‐based evidence of altered mechanical loading or maladaptive subchondral remodelling after anatomically executed MPFL reconstruction.

The persistent inferolateral dominance is biomechanically plausible and reflects long‐term mechanical adaptation. During knee flexion, the resultant quadriceps force vector, in conjunction with trochlear geometry, generates a predominant compressive component directed toward the inferolateral patellar facet [8]. Following Wolff's law and Pauwels' concepts, long‐term subchondral mineralization reflects chronic mechanical adaptation to habitual loading [3, 19]. The preservation of this physiological pattern in both operated and contralateral patellae suggests maintained load transfer rather than pathological redistribution. The absence of medial over‐constraint or asymmetric mineralization is compatible with adequate graft tensioning and anatomically appropriate tunnel placement, although CT‐OAM cannot directly assess these surgical parameters and causality cannot be established.

The present findings align with experimental and computational evidence on patellofemoral contact mechanics after MPFL reconstruction. Biomechanical studies have demonstrated that non‐anatomic tunnel placement or excessive tension increases medial contact pressure and alters tracking [28, 30]. Finite element modelling has similarly shown that non‐physiological reconstruction techniques can increase patellofemoral stress concentrations [22, 23]. In contrast, anatomically executed MPFL reconstruction, with appropriate graft tensioning, aims to restore near‐physiological contact patterns. In the present study, anatomical reconstruction was defined by CT‐based femoral tunnel positioning, and the absence of detectable side‐to‐side asymmetry in the present in vivo data is consistent with a balanced mechanical environment after anatomical reconstruction, although the observational design precludes definitive causal inference.

Previous CT‐OAM studies have established the inferolateral retropatellar density maximum as a characteristic feature of physiological patellofemoral loading in cadaveric specimens [3, 5, 6]. The findings of the present study extend this evidence by demonstrating preservation of this pattern after surgical stabilization in a long‐term follow‐up cohort. The significant interregional differences in HDA fractions (MLF > LLF > CR > MF) confirmed that CT‐OAM can resolve physiological subregional variations in retropatellar loading. The consistency of this gradient across HU thresholds (900, 1000 and 1100) further supports the methodological robustness of the findings within the tested parameter range.

This appears to be the first in vivo long‐term CT‐OAM analysis evaluating subchondral mineralization patterns after isolated MPFL reconstruction. Previous CT‐OAM studies have focused on osteotomy, cartilage degeneration or cadaveric patellae [3, 5, 6, 13]. By combining bilateral in vivo imaging with clinical and functional outcomes, the present study extends prior biomechanical evidence into a patient‐specific long‐term context and demonstrates the potential of CT‐OAM as a complementary tool for characterizing load‐bearing adaptations after joint‐preserving surgery.

Patient demographics and surgical characteristics confirmed appropriate indications for isolated MPFL reconstruction in this cohort. None of the patients exhibited high‐risk patellofemoral factors (Table 1). Clinical examination and PROMs demonstrated good to excellent knee function at follow‐up, with high median scores across all instruments (Table 2). The overall pattern of subjective outcomes was comparable to reports from similar postoperative populations following anatomic MPFL reconstruction [1, 9].

The consistency between favourable clinical outcomes and unchanged subchondral mineralization patterns supports the notion that anatomically executed MPFL reconstruction can maintain physiological patellofemoral loading conditions. However, the cross‐sectional design and small sample size preclude definitive conclusions regarding the relationship between surgical technique, loadings adaptation and long‐term clinical success. The absence of preoperative baseline CT‐OAM data further limits the ability to distinguish surgical effects from pre‐existing patterns.

CT‐OAM characterizes cumulative subchondral mineralization patterns that reflect chronic mechanical adaptation rather than instantaneous stress. This approach complements pressure‐sensor and MRI‐based techniques by depicting the long‐term response of subchondral bone to joint loading. A close spatial relationship between subchondral bone density and local mechanical strength has been demonstrated [5], supporting the biomechanical relevance of CT‐OAM. Owing to its non‐invasive nature and low‐dose CT acquisition protocol, CT‐OAM is particularly suitable for research applications investigating mechanical adaptation after joint‐preserving surgery.

However, CT‐OAM remains an indirect surrogate of joint loading and does not provide direct information on contact pressures, cartilage status or instantaneous force distribution. The interpretation of subchondral density patterns as indicators of loading history relies on the fundamental assumption that bone adapts to mechanical demands according to Wolff's law, which is supported by substantial evidence but may not capture all aspects of patellofemoral biomechanics. The threshold‐based HDA analysis, while methodologically transparent and reproducible across different cutoff values (900–1100 HU), represents a simplified characterization of the continuous density distribution.

To minimize operator‐dependent variability, a semi‐automated workflow was implemented, including standardized registration to a reference patella, edge‐to‐edge bounding box definition using a custom application and automated stripe subdivision with anatomically consistent labelling. Despite these standardization efforts, formal inter‐ and intra‐observer reliability assessment were not performed, which represents an important limitation. The sensitivity analysis across three HU thresholds demonstrated consistent regional patterns and side‐to‐side comparisons, suggesting internal methodological stability within the tested range.

This pilot study has several important limitations that warrant careful consideration. The very small sample size (*n* = 9) limits statistical power and increases the risk of Type II error, precluding definitive conclusions about the absence of side‐to‐side differences. The findings should therefore be interpreted as exploratory and hypothesis‐generating rather than confirmatory. The retrospective cross‐sectional design precludes longitudinal assessment of loading adaptation over time and does not allow differentiation between surgical effects and pre‐existing patterns due to the absence of preoperative baseline CT‐OAM data. Femoral tunnel position and graft tension were not directly measured or correlated with CT‐OAM findings. These surgical parameters can only be presumed based on operative technique and clinical outcomes. Formal inter‐ and intra‐observer reliability assessments for image processing and regional segmentation were not performed, which limits generalizability of the measurement approach despite the implementation of standardized procedures. Manual adjustment of projection axis and region‐of‐interest definition may introduce operator‐dependent variability, although the semi‐automated workflow was designed to minimize such effects. CT‐OAM serves as an indirect surrogate of loading without direct correlation to cartilage status, in vivo contact pressure measurements or kinematic data.

Despite these limitations, the consistent results across HU thresholds and the reproducible regional rank order lend internal validity to the analysis.

## CONCLUSION

At a minimum of 7.5 years after anatomically executed, isolated MPFL reconstruction, retropatellar subchondral mineralization patterns showed no statistically significant side‐to‐side differences between operated and contralateral patellae, and the regional distribution was consistent with physiological loading. While these findings provide no CT‐OAM‐based evidence of altered long‐term mechanical adaptation or pathological medial overload, the small sample size precludes definitive conclusions about equivalence due to the risk of Type II error. CT‐OAM demonstrated feasibility for characterizing cumulative subchondral responses after patellofemoral stabilization surgery and may serve as a useful adjunct for evaluating long‐term load‐bearing patterns following joint‐preserving procedures.

## AUTHOR CONTRIBUTIONS

All authors have read and agreed to the published version of the manuscript.

## CONFLICT OF INTEREST STATEMENT

The authors declare no conflicts of interest.

## ETHICS STATEMENT

The study was approved by the Ethics Committee of the Medical University of Innsbruck (EK Nr. 1114/2021) and conducted in accordance with the Declaration of Helsinki. Informed consent was obtained from the patient involved in the study.

## Supporting information

Supporting File

## Data Availability

The data presented in this study are available on request from the corresponding author. The data are not publicly available due to privacy reasons.
