# Supplementary material for: No significant side‐to‐side differences in retropatellar load distribution more than 7.5 years after isolated MPFL reconstruction: A CT‐osteoabsorptiometry pilot study in nine patients
Source: J Exp Orthop. 2026 May 4;13(2):e70728. doi: 10.1002/jeo2.70728 (PMC13137781; doi:10.1002/jeo2.70728)
Supplement: Supplementary file 1 — Supporting File [file JEO2-13-e70728-s001.docx]

**Supplementary**

**Figure A1.** Maximum intensity projections of CT-osteoabsorptiometry for all included patients, depicting subchondral mineralization patterns of the left and right patellae. Each row shows the paired patellae of one patient. The side label shown in red to indicate the operated side.


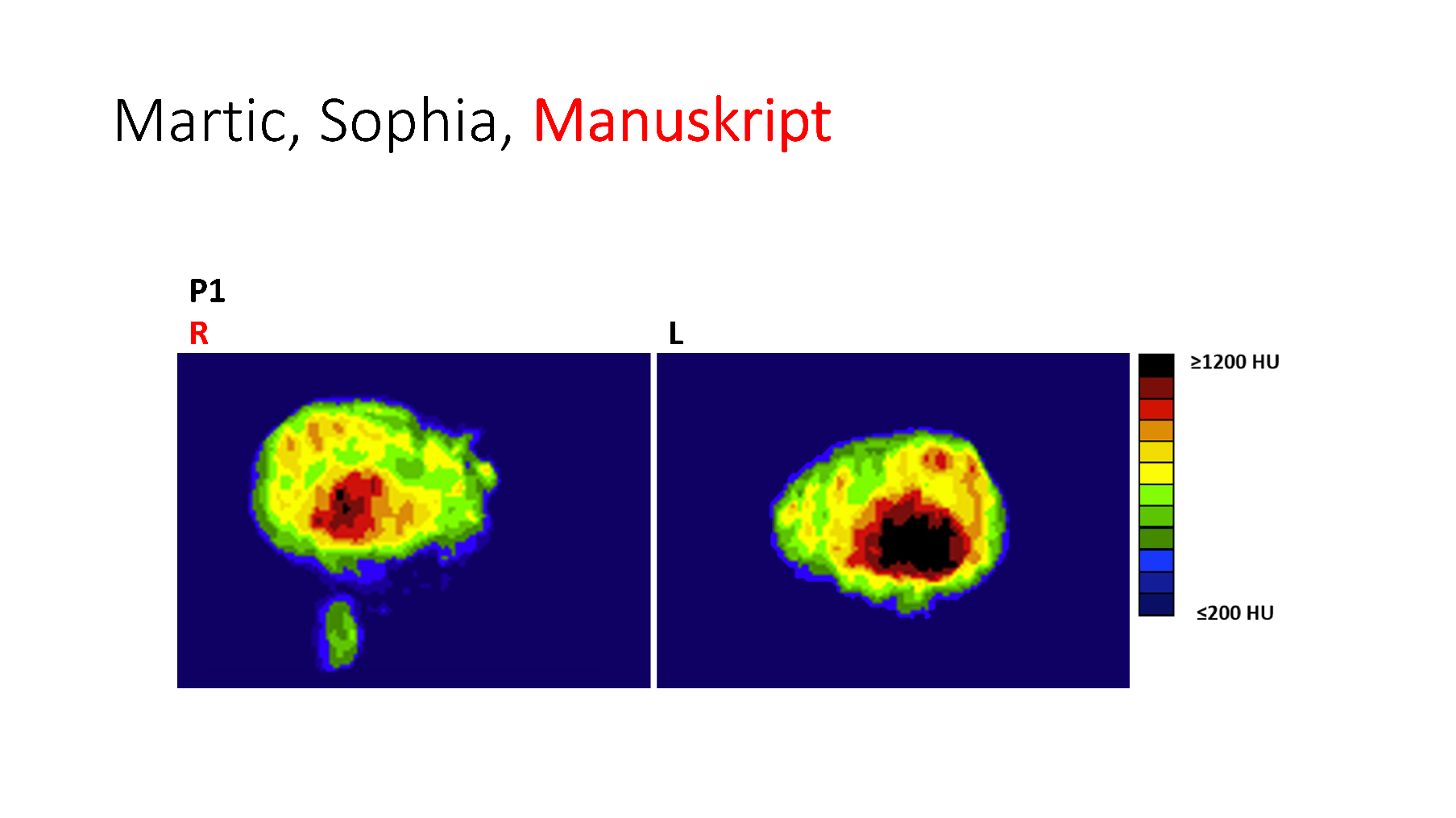


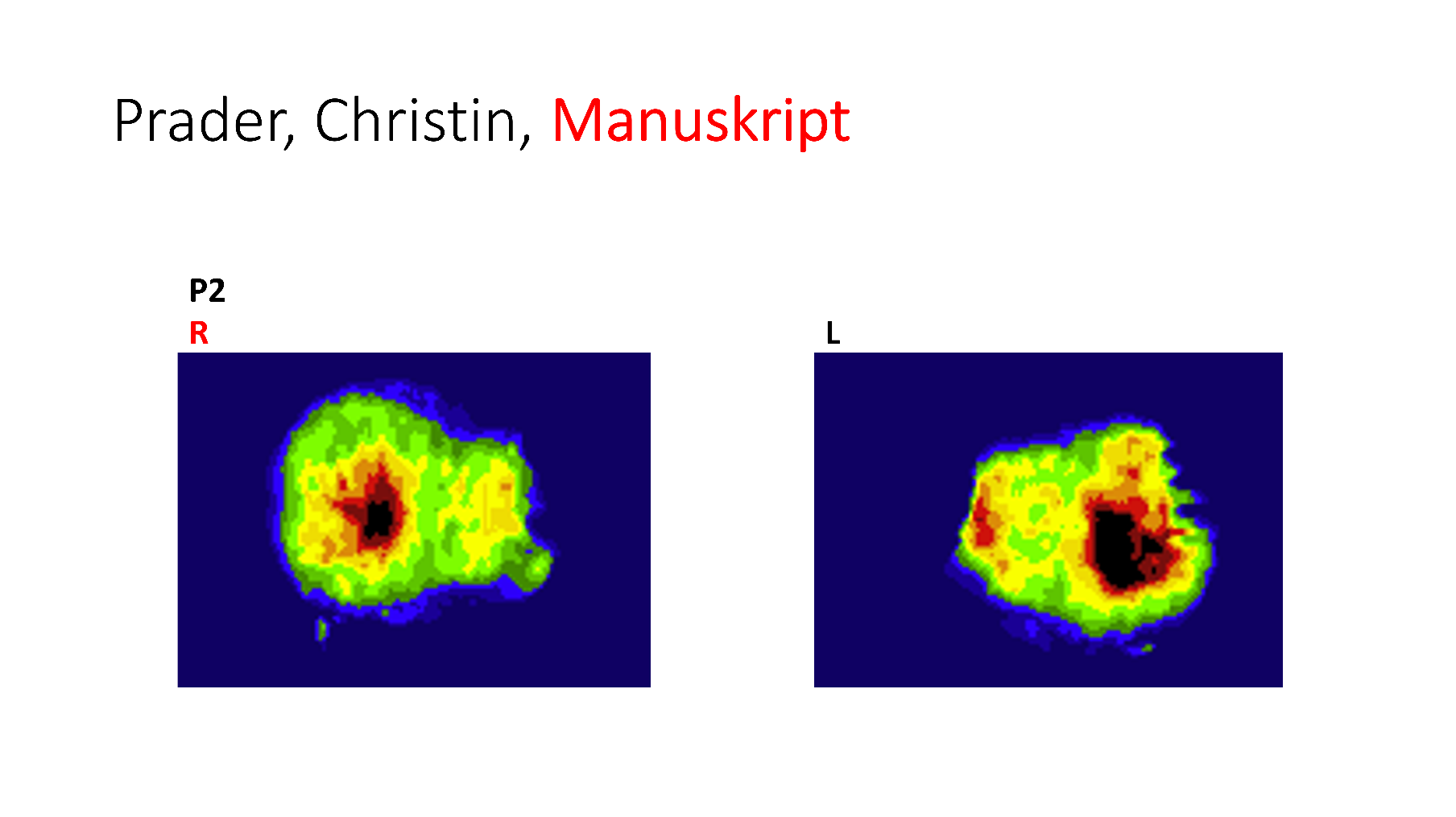


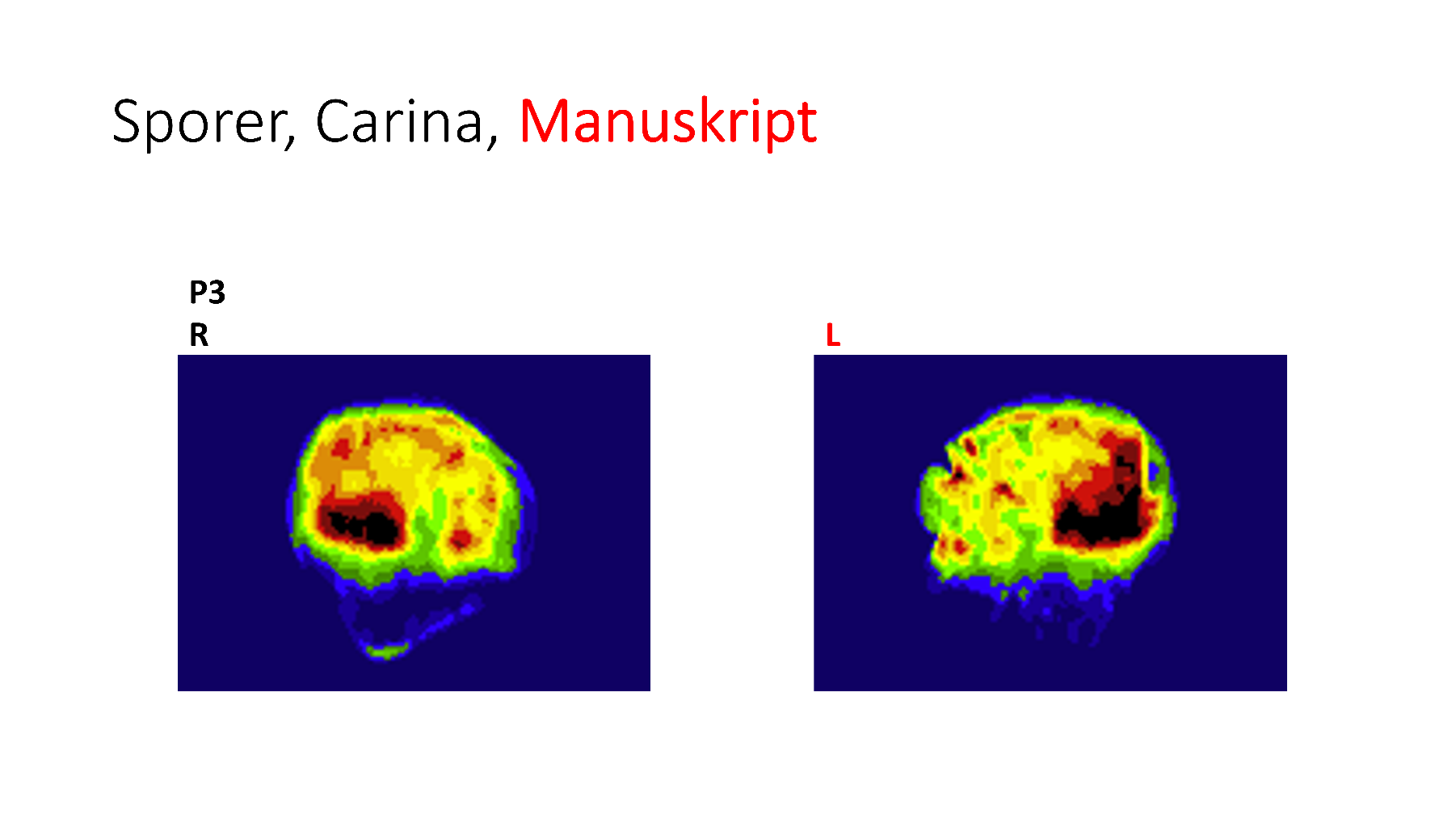


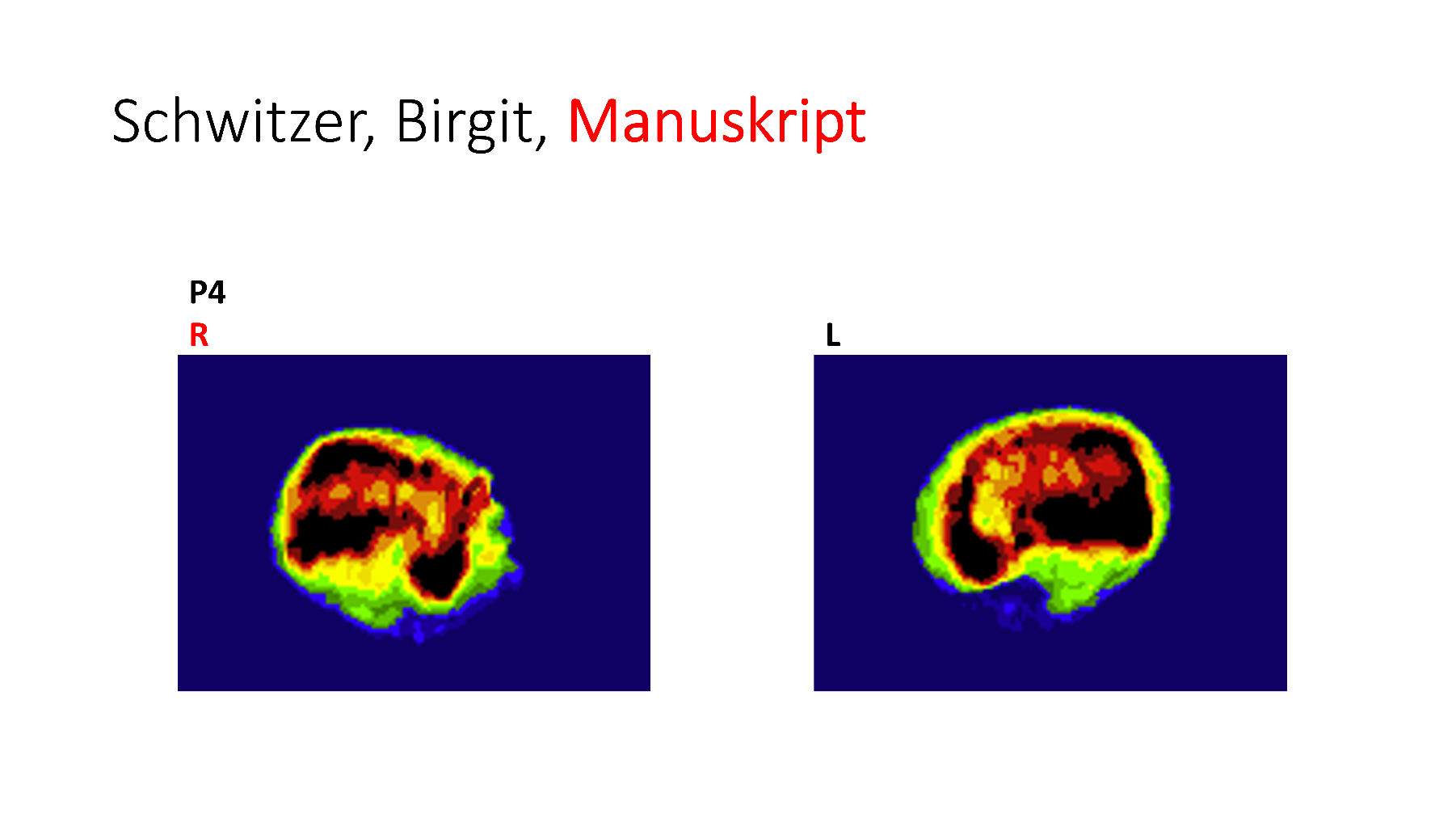


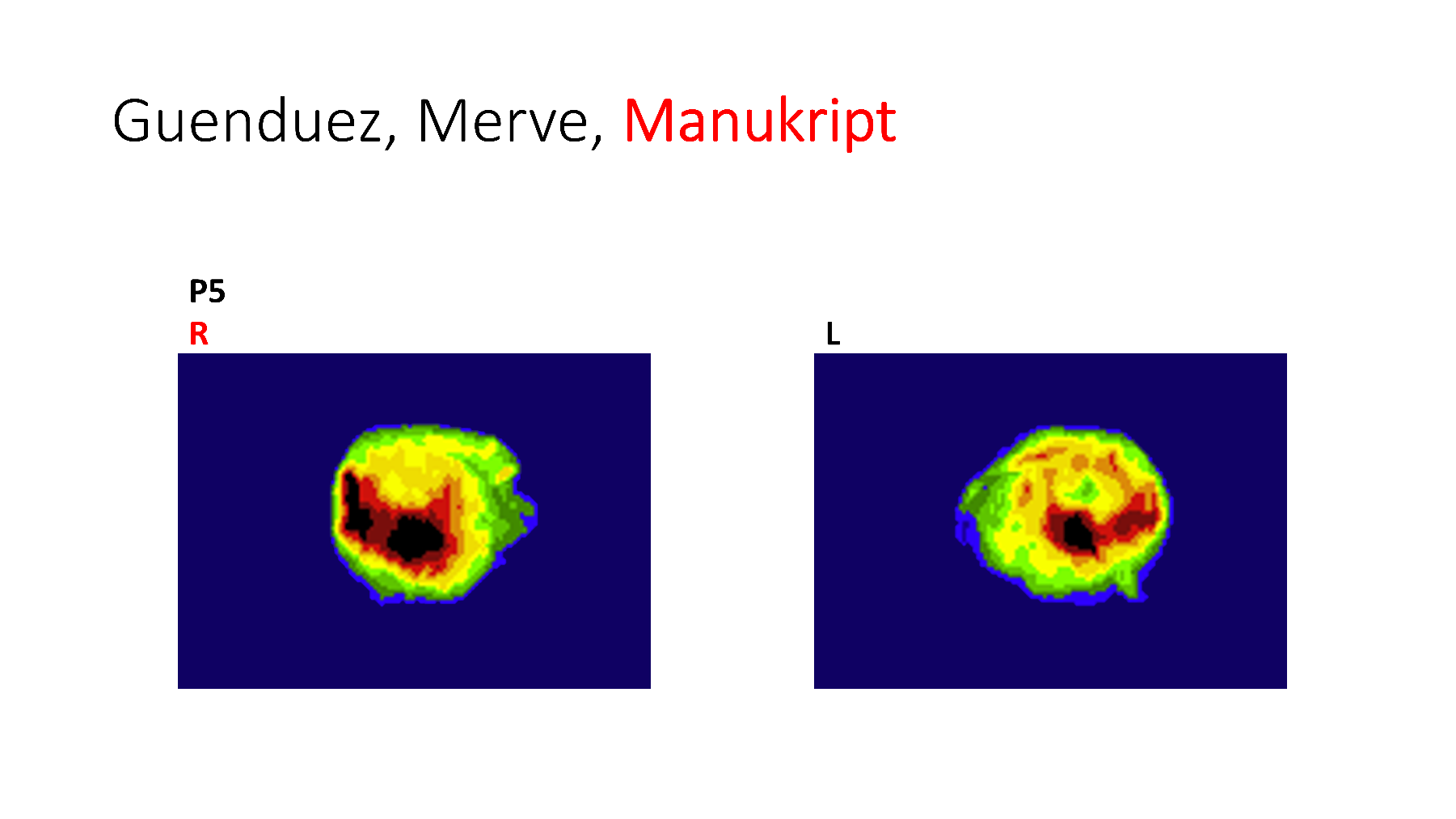


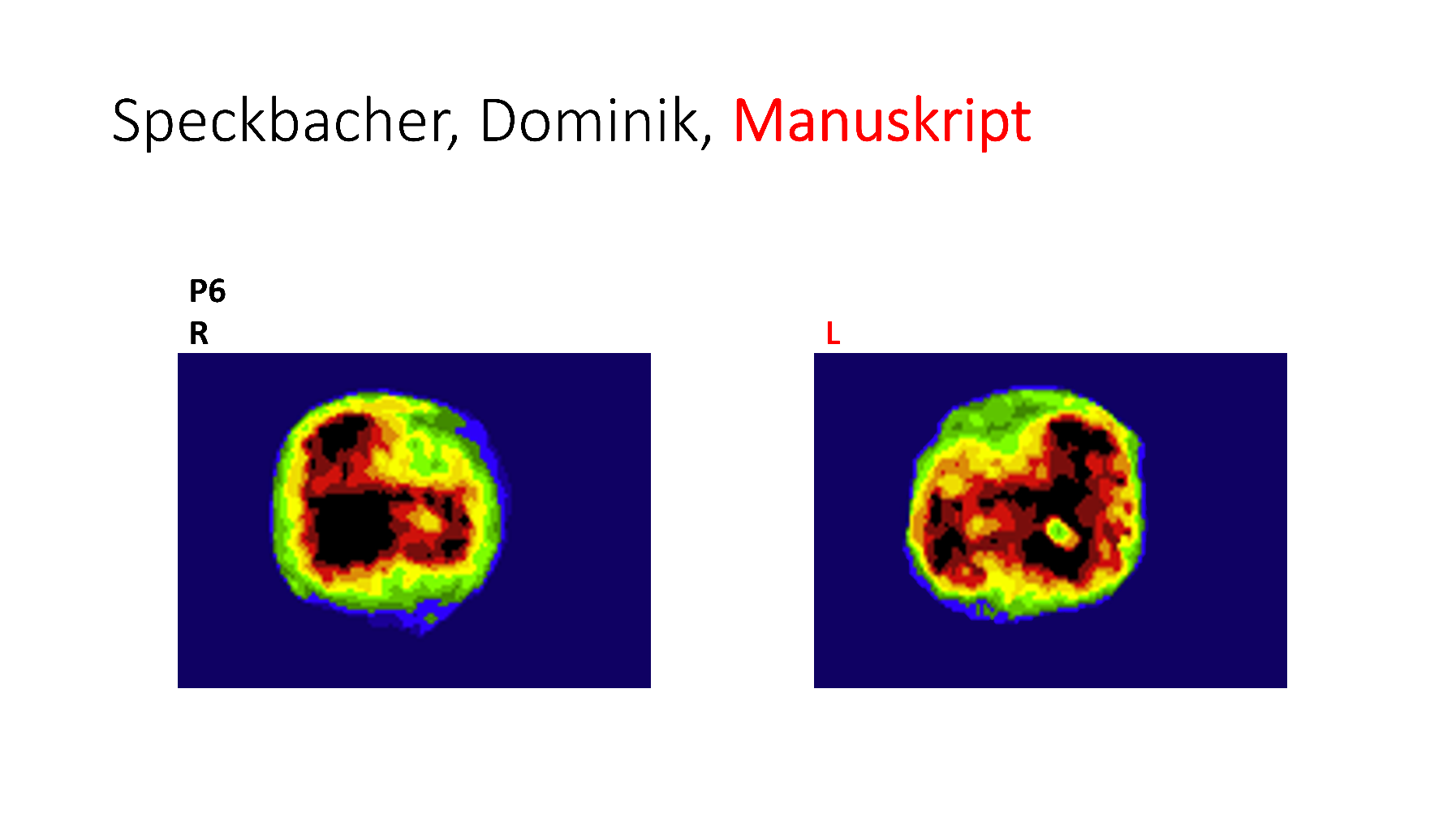


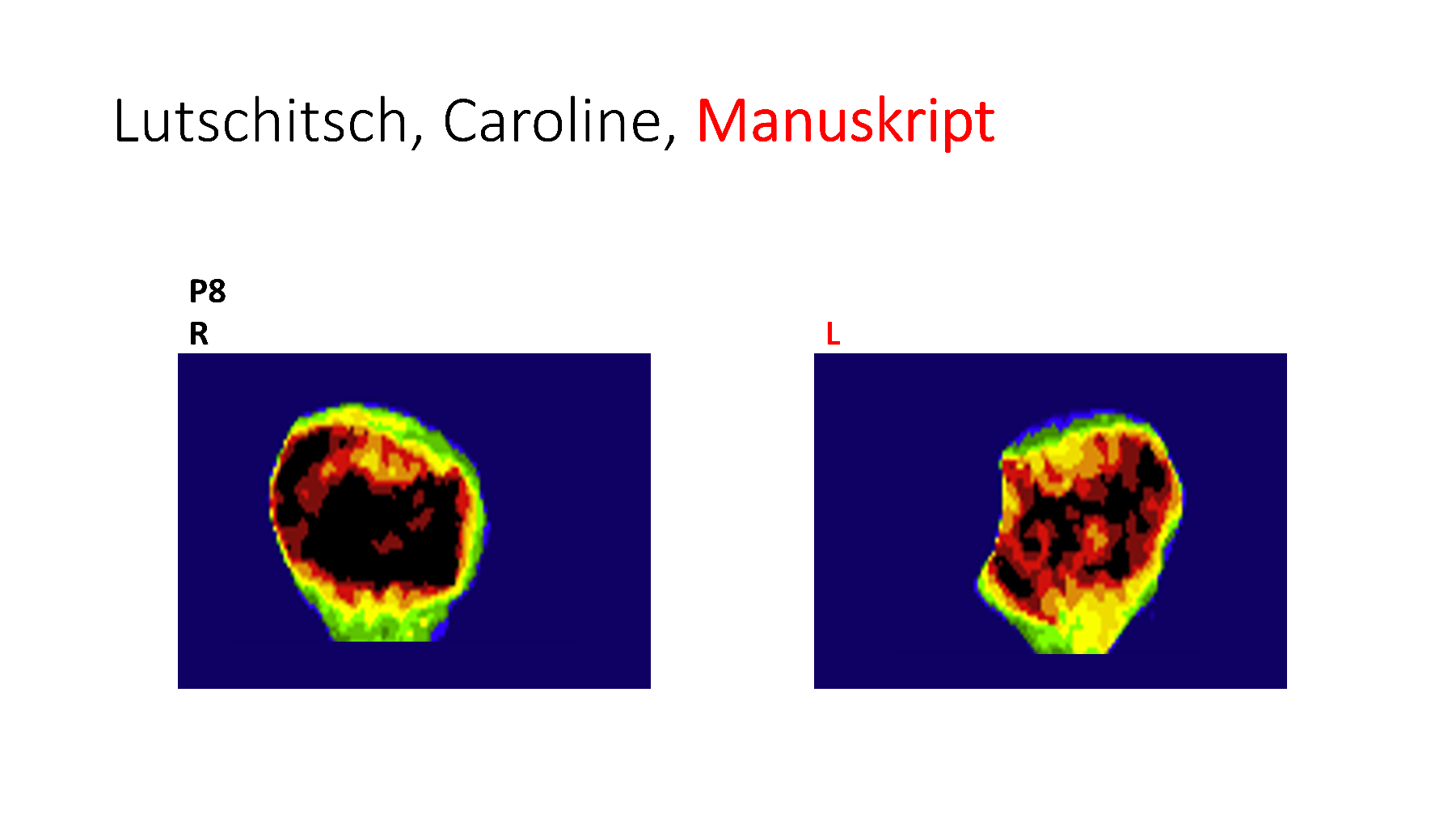


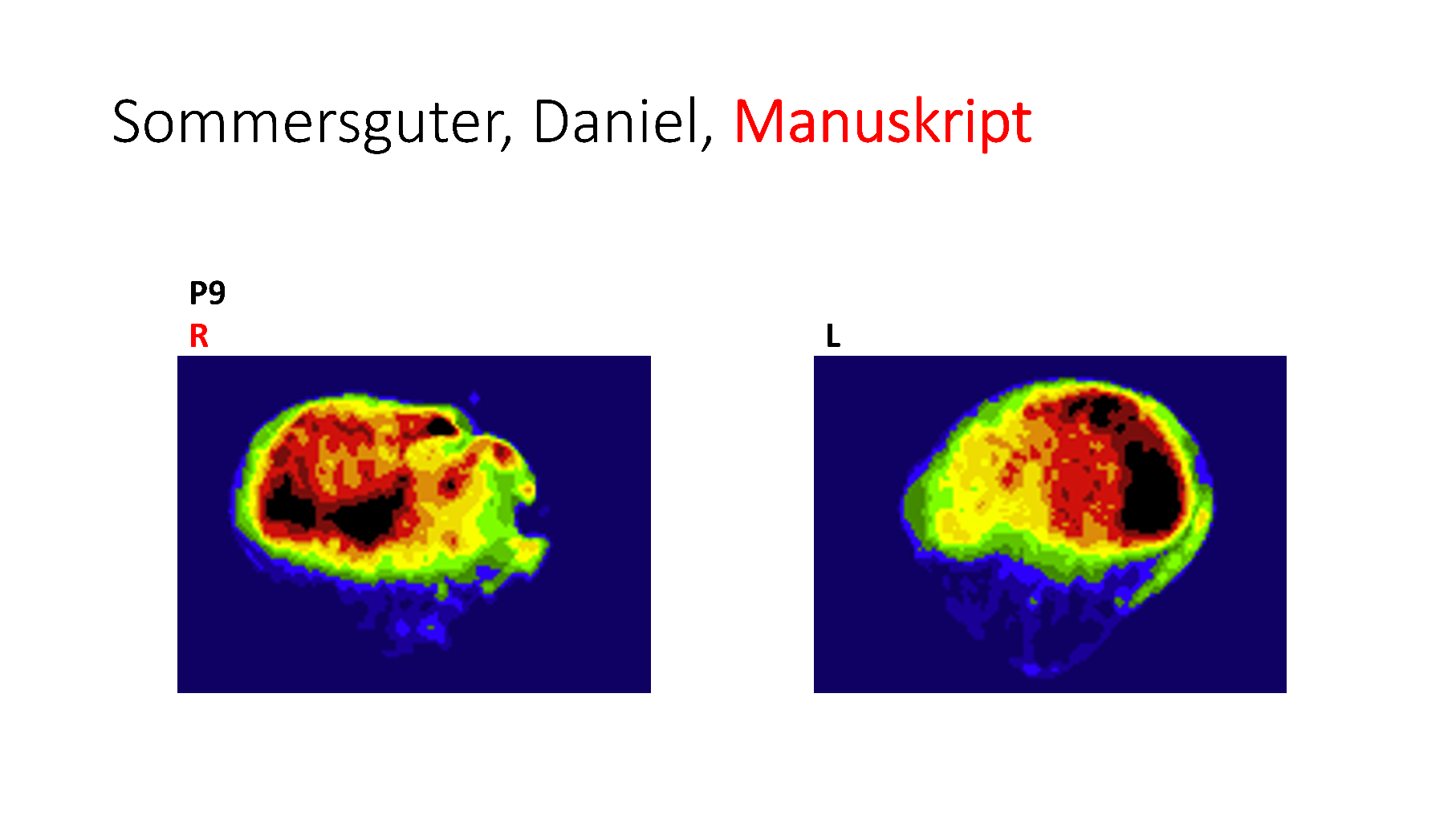


HU, Hounsfield unit; L, left side; P, patient; R, right side.**
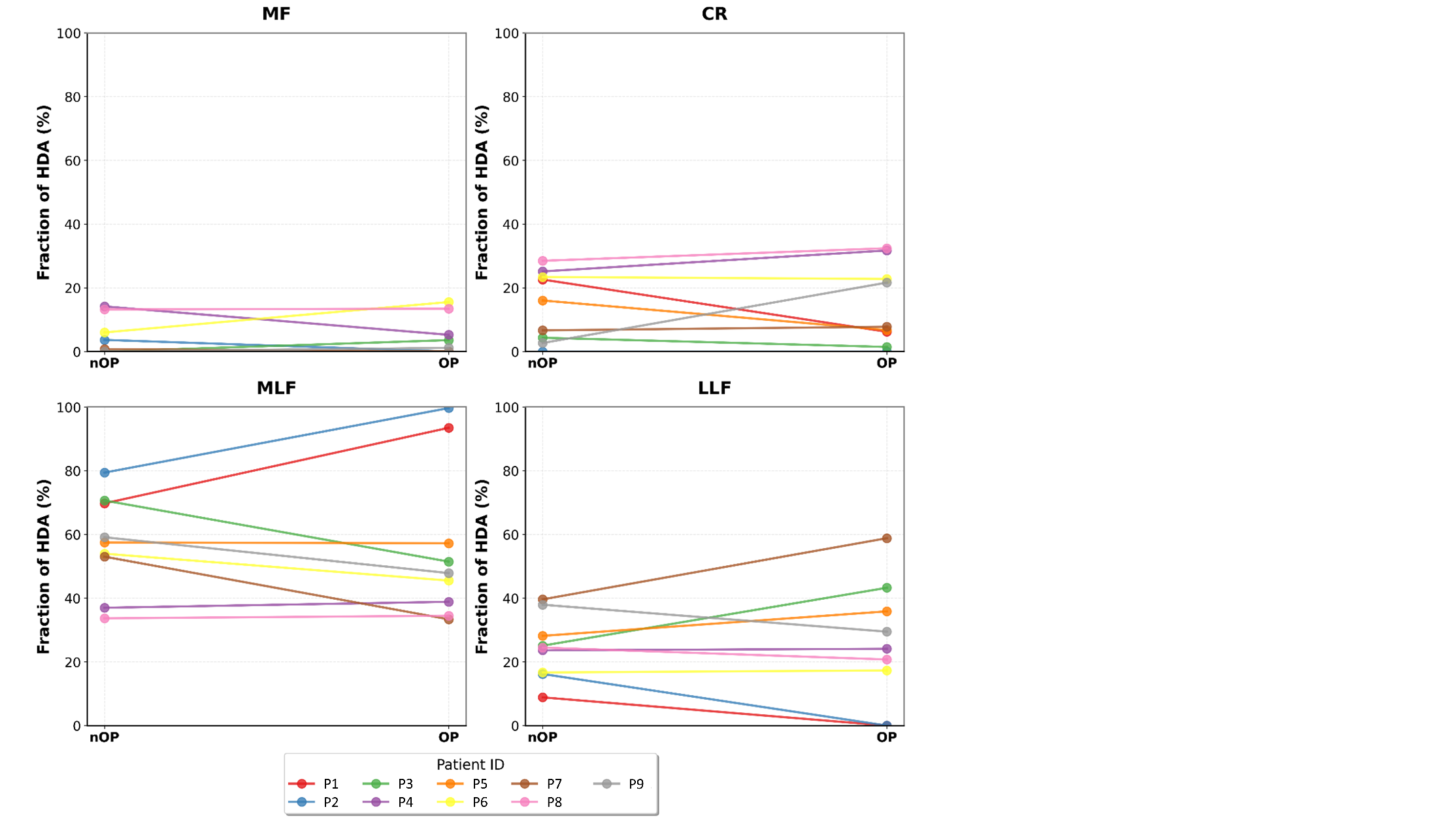
**

**Figure A2.** Individual patient changes of HDA fractions across patellar regions (n = 9, HU threshold = 1000). Each colored line represents one patient, connecting non-operated (nOP) to operated (OP) knee for the four patellar stripe regions.

CR, central ridge; HDA, high-density area; HU, Hounsfield units; LLF, lateral portion of the lateral facet; MF, medial facet; MLF, medial portion of the lateral facet; nOP, non-operated; OP, operated.

**Supplementary Table A1.** Patient-level HDA fractions (%) for nOP and OP sides across patellar regions.

| Patient | Follow-up (years) | Side | Fraction of HDA [%] | | | |
| --- | --- | --- | --- | --- | --- | --- |
|  |  |  | MF | CR | MLF | LLF |
| P1 | 12,3 | nOP | 0 | 22,6 | 69,7 | 8,9 |
|  |  | OP | 0 | 6,2 | 93,5 | 0 |
|  |  | OP - nOP | 0 | -16,4 | 23,8 | -8,9 |
| P2 | 10,4 | nOP | 3,6 | 0 | 79,4 | 16,2 |
|  |  | OP | 0 | 0 | 99,7 | 0 |
|  |  | OP - nOP | -3,6 | 0 | 20,3 | -16,2 |
| P3 | 8,3 | nOP | 0 | 4,3 | 70,6 | 25,1 |
|  |  | OP | 3,6 | 1,4 | 51,5 | 43,3 |
|  |  | OP - nOP | 3,6 | -2,9 | -19,1 | 18,2 |
| P4 | 8,9 | nOP | 14,2 | 25,1 | 37 | 23,6 |
|  |  | OP | 5,2 | 31,7 | 38,9 | 24,1 |
|  |  | OP - nOP | -9 | 6,6 | 1,9 | 0,5 |
| P5 | 11,3 | nOP | 0 | 16 | 57,5 | 28,2 |
|  |  | OP | 0 | 6,7 | 57,2 | 35,9 |
|  |  | OP - nOP | 0 | -9,3 | -0,3 | 7,7 |
| P6 | 9 | nOP | 6 | 23,4 | 54 | 16,7 |
|  |  | OP | 15,5 | 22,8 | 45,5 | 17,3 |
|  |  | OP - nOP | 9,5 | -0,6 | -8,5 | 0,6 |
| P7 | 11 | nOP | 0,8 | 6,6 | 53 | 39,6 |
|  |  | OP | 0 | 7,8 | 33,4 | 58,8 |
|  |  | OP - nOP | -0,8 | 1,2 | -19,6 | 19,2 |
| P8 | 7,7 | nOP | 13,2 | 28,5 | 33,7 | 24,5 |
|  |  | OP | 13,4 | 32,4 | 34,5 | 20,7 |
|  |  | OP - nOP | 0,2 | 3,9 | 0,8 | -3,8 |
| P9 | 8,3 | nOP | 0 | 2,7 | 59,1 | 37,9 |
|  |  | OP | 1,1 | 21,6 | 47,9 | 29,5 |
|  |  | OP - nOP | 1,1 | 18,9 | -11,2 | -8,4 |

CR, central ridge; HDA, high-density area; HU, Hounsfield units; LLF, lateral portion of the lateral facet; MF, medial facet; MLF, medial portion of the lateral facet; nOP, non-operated; OP, operated.

**Table A2.** Sensitivity analysis of HU thresholds (900 and 1100) for HDA fractions. Values are medians [IQR] for operated and contralateral non-operated patellae. Holm-adjusted p-values (Wilcoxon signed-rank) and corresponding effect sizes (rank-biserial correlation, r) are reported for each region. No statistically significant differences were detected at any threshold (all adjusted p > 0.05).

| **HU- level** | **Strip** | **nOP** | | **Op** | | **p-adjusted** | **Rank-biserial r** |
| --- | --- | --- | --- | --- | --- | --- | --- |
|  |  | **Median [%]** | **IQR [%]** | **Median [%]** | **IQR [%]** |  |  |
| 900 | MF | 2.0 | 0.0-6.2 | 1.4 | 0.0-6.1 | 1 | 0.04 |
|  | CR | 16.4 | 7.4-24.2 | 12.0 | 6.9-24.3 | 1 | 0.04 |
|  | MLF | 55.4 | 52.4-66.8 | 48.7 | 38.5-53.8 | 1 | 0.06 |
|  | LLF | 23.8 | 17.0-29.7 | 23.1 | 18.1-35.9 | 1 | 0.06 |
| 1100 | MF | 0.0 | 0.0-5.9 | 1.1 | 0.0-4.9 | 1 | 0.16 |
|  | CR | 16.3 | 2.2-22.2 | 5.2 | 4.3-21.8 | 1 | 0.08 |
|  | MLF | 56.8 | 54.1-71.5 | 47.4 | 38.9-59.0 | 1 | 0.14 |
|  | LLF | 24.8 | 18.7-31.4 | 22.8 | 16.6-34.6 | 1 | 0.02 |

CR, Central ridge; HDA, High-Density Area; HU, Hounsfield Units; IQR, Interquartile range; LLF, Lateral portion of the lateral facet; MF, Medial facet; MLF, Medial portion of the lateral facet; nOP, Non-operated; OP, Operated; p-adjusted, Holm-adjusted p-value; r, rank-biserial correlation.
